# Seroprevalence of *Neospora caninum* in pet cats, dogs and rabbits from urban areas of Poland

**DOI:** 10.1186/s12917-024-03894-3

**Published:** 2024-01-31

**Authors:** Hanna Turlewicz-Podbielska, Jakub Jędrzej Ruszkowski, Jarosław Wojciechowski, Małgorzata Pomorska-Mól

**Affiliations:** 1 Department of Preclinical Sciences and Infectious Diseases, Poznan University of Life Sciences, Wolynska 35, 60‑637, Poznan, Poland; 2 Department of Animal Anatomy, Faculty of Veterinary Medicine and Animals Sciences, Poznan University of Life Sciences, Wojska Polskiego 71C, 60-625 Poznan, Poland; 3 VETPOL Sp. z o.o, Grabowa 3, Grudziadz, 86-300 Poland

## Abstract

**Background:**

*Neospora caninum* (*N. caninum*) has a broad intermediate host range and might cause multisystemic lesions in various species of animals. Dogs are both intermediate and definitive hosts of the parasite and play a crucial role in the horizontal transmission of this protozoan to other animals. Cats and rabbits could be sensitive to infection with *N. caninum*, however, clinical symptoms and the exact route of infection in these species are unknown. The epidemiology of *N. caninum* in cats and rabbits has been barely researched, and there is no published record of the seroprevalence of *N. caninum* infection in these species in Poland. Thus, the present study aimed to determine the frequency of seroreagents for *N. caninum* within pet dogs, cats and rabbits from urban areas of Poland and to identify possible risk factors for these animals.

**Results:**

In total, serum samples from 184 cats (*Felis catus*), 203 dogs (*Canis familiaris*) and 70 rabbits (*Oryctolagus cuniculus*) were used in the study. The seroprevalence of anti-*N. caninum* antibodies in dogs and cats reached 1.0% (2/203; 95% CI: 0.3–3.5) and 3.3% (6/184; 95% CI: 1.5–6.9), respectively. No significant differences in seroprevalence regarding age group, gender, symptoms or sampling location were found. All 70 samples from pet rabbits were negative for anti-*N. caninum* antibodies.

**Conclusions:**

The seroprevalence rates of *N. caninum* in dogs and cats in the present study were low, however, our results confirmed *N. caninum* circulates among dog and cat populations in Poland, and neosporosis should be included in the differential diagnosis of neuro-muscular disorders in these species. This is the first serological survey of *N. caninum* in European pet cats and rabbits. The role of pet rabbits in *N. caninum* epidemiology and circulation in Poland is marginal.

## Background

*Neospora caninum* (*N. caninum*) is an obligate intracellular apicomplexan parasite that might cause multisystemic lesions in dogs and other species [1]. Dogs are both intermediate and definitive hosts of the parasite and play a crucial role in the horizontal transmission of this protozoan to other animals [2, 3]. *N. caninum* has a broad intermediate host range [3]. After ingestion of sporulated oocysts by the intermediate hosts, sporozoites are released from oocysts and converted into tachyzoites, which disseminate the infection. The horizontal transmission includes ingestion of sporulated oocysts or infected tissues of intermediate hosts with tissue cysts [3]. The cats could be sensitive to infection with *N. caninum*, but clinical symptoms and the exact route of infection are unknown [4]. The clinical form of neosporosis is characterized by the onset of neuromuscular symptoms [5]. However, neosporosis is often missed in diagnosing neurological disorders and myopathies in dogs and cats. The serological studies regarding *N. caninum* in domestic animals usually focus on dogs [6–13]. Several studies were conducted to determine the seroprevalence of *N. caninum* in domestic cats worldwide [13–16]. Data on *N. caninum* seroprevalence in rabbits are scarce, although few reports indicate the presence of antibodies against *N. caninum* in rabbit sera [12, 17, 18]. The epidemiology of *N. caninum* in cats and rabbits has been barely researched, and there is no published record of the seroprevalence of *N. caninum* infection in these species in Poland. Thus, the present study aimed to determine the frequency of seroreagents for *N. caninum* within pet dogs, cats and rabbits from urban areas of Poland and to identify possible risk factors for these animals.

## Results

Anti-*N. caninum* antibodies were found in sera collected from dogs (*Canis familiaris*) and cats (*Felis catus*). In dogs and cats, the overall seroprevalence was 1.0% (2/203; 95% CI: 0.3–3.5), and 3.3% (6/184; 95% CI: 1.5–6.9), respectively. All of the serum samples from rabbits (*Oryctolagus cuniculus*) were negative for antibodies to *N. caninum*. No significant gender difference in seroprevalence between females and males and no significant differences regarding age group, symptoms, or sampling location were found (Table 1).

**Table 1:** Seropositivity among cats and dogs split into risk factor groupings and detailed information about the structure of the sampled rabbit population

			Dogs				Cats			Rabbits
		(Positive) Total	Prevalence (%)	95% CI^a^	p-value	(Positive) Total	Prevalence (%)	95% CI^a^	p-value	No. of ind.^b^
Age (years)										
	< 1	(0) 23	0.0	0.0-14.3	0.97	(1) 22	4.6	0.8–21.8	0.46	16
	1–3	(0) 35	0.0	0.0-9.9		(3) 46	6.5	2.2–17.5		23
	4–7	(1) 44	2.3	0.4–11.8		(1) 45	2.2	0.4–11.6		22
	8+	(1) 101	1.0	0.2–5.4		(1) 71	1.4	0.3–7.6		7
	No data	-				-				2
Sex										
	F	1 (103)	1.0	0.2–5.3	0.99	(2) 87	2.3	0.6-8.0	0.78	40
	M	1 (100)	1.0	0.2–5.5		(4) 97	4.1	1.6–10.1		30
Location										
	Deblin	(0) 26	0.0	0.0-12.9	0.21	(0) 11	0.0	0.0-25.9	0.83	-
	Kluczbork	(0) 22	0.0	0.0-14.9		(0) 7	0.0	0.00-35.4		-
	Lublin	(0) 47	0.0	0.0-7.6		(2) 58	3.5	0.95–11.7		-
	Poznan	(1) 93	1.1	0.2–5.8		(4) 92	4.5	1.70–10.7		70
	Przemysl	(1) 14	7.1	1.3–31.5		(0) 16	0.0	0.00-19.4		-
Symptoms										
	Neurological disorders	(0) 8	0.0	0.0-32.4	0.61	(1) 11	9.1	1.6–37.7	0.48	2
	Other	(2) 136	1.5	0.4–5.2		(3) 120	2.5	0.9–7.1		22
	Healthy	(0) 59	0.0	0.0-6.1		(2) 53	3.8	1.0-12.8		46
Total		(2) 203	1.0	0.3–3.5		(6) 184	3.3	1.5–6.9		70

^a^95% CI: lower and upper values for the 95% confidence interval; ^b^Number of individuals

## Discussion

The antibodies against *N. caninum* in the cats and dogs indicate susceptibility to infection and induction of an immune response. In previous studies conducted in Poland, antibodies against *N. caninum* were detected in 21.7% (56/257) of dogs [7]. Antibodies against *N. caninum*, with various prevalence, have also been detected previously in dogs in several European countries: Spain (51% in farm dogs (51/100) and 2.9% in pet dogs (3/102)) [6], in Germany in breeding bitches (7.33%; 16/218) [9] and in dogs in Portugal (32.5%; 93/286) [12] and Türkiye (16.6%; 21/187) [8]. The seroprevalence in dogs obtained in our study was low compared to other European countries or studies previously conducted in Poland and reached 0.99% (95% CI: 0.27–3.52). Serological studies conducted on cats in several European countries reported seroprevalence similar to our results: Czech Republic (3.86%; 16/414) [16], Spain (6.8%, 4/59) [15] and Hungary (0.6%; 2/330) [14]. The lower rates of seropositivity to *N. caninum* in pets than in stray or farm animals might be due to dietary habits, as outdoor animals have direct contact with infective material from positive cattle or other intermediate hosts, and they have common access to contaminated environments [19]. All dogs and cats included in the investigation came from an urban area and were kept at home, and the likelihood of their contact with sporulated oocysts or infected tissues is low. Still, as revealed, an infection is not impossible. Pet animals living in cities may come into contact with a potentially infected environment while walking or travelling. Cats that are temporarily left outdoors unattended or have escaped may also have the opportunity to acquire the infection with *N. caninum*. Additionally, feeding with infected raw meat, placenta, fetuses or uterine discharge from ruminants may be a potential infection risk factor for pet carnivores [20, 21]. Villagra-Blanco confirmed 6 (37.5%) of the 16 seropositive bitches found in the study (7.3%; 16/218) were fed raw diets [9]. Our research shows that despite not living a typical outdoor lifestyle, there is still a risk of infection in pet dogs and cats. To date, two articles have been published on the seroprevalence of *N. caninum* in rabbits in European countries. However, the studies were conducted on the wild rabbit (seroprevalence 25%, 8/32) [12] and farm rabbits (seroprevalence 1.2%, 3/260) [18]. The available data on the *N. caninum* seroprevalence in pet rabbits are scarce - there is one report from Japan, although, Western blot and the indirect immunofluorescence assay did not reveal any positivity in 337 serum samples [17]. In our study, we also did not detect anti-*N. caninum* antibodies in pet rabbits. These results confirmed that the possibility of ingesting infective *N. caninum* oocysts by pet rabbits (via unwashed plants, hay) is negligible, and domestic rabbits probably do not play an important role in the epidemiology of *N. caninum* infections.

In most cases, neosporosis is asymptomatic in adult dogs [5]. However, this disease should be considered when hyperesthesia, atrophy or muscle swelling are revealed at the clinical exam [5]. Primary clinical neosporosis usually occurs as a neuro-muscular form of the disease, although protozoan tachyzoite proliferation occurs in many tissues, and the symptoms and clinical picture can differ [5, 20]. Nevertheless, seropositive dogs in our study showed no neurological abnormalities at sampling and in the past. Moreover, there are no reports of natural infection of cats with *N. caninum* in the literature. According to Dubey et al. [4], cats are susceptible to experimental infection, and they may develop neuro-muscular disorders after infection. One of the seropositive cats showed neurological disorders at the time of sampling; however, the statistical analysis of our results indicates no significant association between health status and seropositivity in both dogs and cats. This may be because the clinical picture depends on many variables, such as the immune status of the host and the site of tachyzoite proliferation. Rabbits can acquire *N. caninum* infection through the ingestion of sporulated oocysts of the aforementioned parasites and through congenital transmission from rabbit dams to their fetuses [17]. However, clinical neosporosis in rabbits was not reported in the literature to date.

Our results indicate no significant differences between seropositivity and gender in dogs and cats and are consistent with those obtained in previous European studies [6, 14]. No significant differences between seropositivity in dogs and cats and age have also been found in our study. Results for dogs are consistent with those obtained by Goździk et al. [7] and Collantes-Fernández et al. [6] Collantes-Fernández et al. [6] noted a significant increase in seropositivity with age only in farm dogs. However, Hornok et al. [14] observed a significant increase in the presence of anti-*N. caninum* antibodies with age in cats. No significant differences between seropositivity and sampling city have been observed in the present study.

The present study has some limitations that should be kept in mind. The most important serological tests used in the laboratory diagnostics of neosporosis are enzyme-linked immunosorbent assay (ELISA) and immunofluorescent antibody test. Both have a high sensitivity and specificity [10]. We have searched for evidence of *N. caninum* infection via one serological test (indirect ELISA). In addition, the cross-reactivity to the related pathogens e.g. *Toxoplasma gondii* cannot be ruled out despite the high specificity of the ELISA used in the present study.

## Conclusion

Our results update the canine *N. caninum* seropositivity data in Poland and provide new data on the epidemiology of neosporosis in this species. The present study is the first serological survey of *N. caninum* in European pet cats and rabbits. Although this study found a low positive rate of *N. caninum* in dogs and cats in urban areas, more attention is needed to prevent the transmission between definitive hosts and accidental hosts. *N. caninum* probably circulates among dog and cat populations in Poland, and neosporosis should be included in the differential diagnosis of neuro-muscular disorders in these species. The role of pet rabbits in *N. caninum* epidemiology and circulation in Poland is marginal.

## Methods

### Samples

**Fig. 1:**
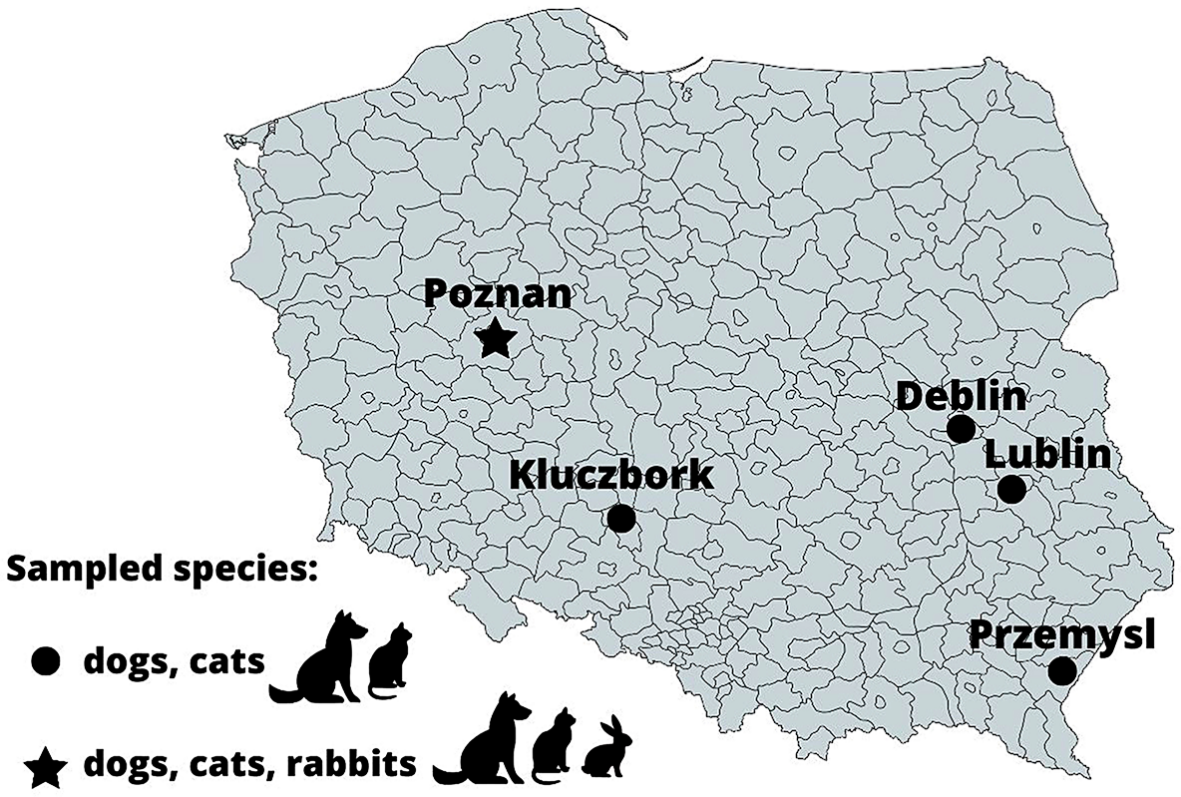
Map of sampling regions and species from which samples were collected

A total of 467 serum samples collected between September 2020 and January 2022 in five veterinary practices located in various parts of Poland (Poznan 52°24′24″N 16°55′47″E (wielkopolskie voivodeship); Przemysl 49°47′05″N 22°46′02″E (podkarpackie voivodeship); Kluczbork 18°13′E 50°58′N (opolskie voivodeship); Lublin 22°34′E 51°15′N (lubelskie voivodeship); Deblin 21°52′E 51°34′N (lubelskie voivodeship) (Fig. 1.) were selected for this study. Samples from rabbits were collected in one practice (*n* = 70). Rabbit sera came from animals from Poznan and their surroundings. Sera were stored at − 70 °C until analyses. In total, serum samples from 184 cats, 203 dogs and 70 rabbits were randomly selected and used in the study. For each sample, the following information has been available: species, gender, age at sampling, location, and represented symptoms (neurological disorders, others, healthy). The detailed information about the structure of a sampled population is presented in Table 1.

### Detection of *N. caninum* antibodies

Due to the multi-species nature and high specificity and sensitivity, a competitive ELISA has been used to perform serological testing. Serum samples were tested for the presence of antibodies against *N. caninum* by using the ID Screen® *Neospora caninum* competition (IDvet, Grabels, France) according to the manufacturer’s instructions. The commercial ELISA kit was validated for the detection of anti-*N. caninum* antibodies in serum or plasma from ruminants, dogs, or other susceptible species. Briefly, 50 µL of sera was diluted with 50 µL ELISA dilution buffer in the test plate and incubated for 45 min at 37°C in a humid chamber. The plate was washed three times, and 100 µL of the conjugate was added to each well. Then, the plate was incubated for 30 min at room temperature (21°C +/- 5°C) and washed three times. Next, 100 µL of 3,3’,5,5’-tetramethylbenzidine substrate solution was added to each well for 15 min, followed by 100 µL of stop solution. The optical density (OD) was measured at 450 nm in the Infinite® 200 PRO microplate reader (TECAN) immediately after stopping the reaction. The results were calculated as the percentage S/N (S/N%) for each test sample according to the following formula: S/N (%) = OD sample/OD negative control × 100. If the S/N% was less than 50, the sample was considered positive, < S/N% ≤ 60 indicated a doubtful result and > 60 S/N% a negative result. Specific details of the ELISA kits, along with the sensitivities and specificities of the assays, are 100% (IC 95%: 98.8–100%) and 100% (CI 95%: 99.41–100%), respectively [22].

### Statistical analysis

The analyses were performed using RStudio (version 4.1.2), except for prevalence (with 95% confidence intervals (CI)), which was available as an online program (https://epitools.ausvet.com.au/ciproportion). CI for prevalence was calculated with the Wilson score method. Pearson’s chi-square (*χ*2) tests were used to analyse the data in different age groups, locations, genders and health status. A *p*-value of < 0.05 was considered significant. The map was created using an online program Map Chart (https://www.mapchart. net) and Canva (https://www.canva.com).

## Data Availability

The data used to support the findings of this study are available from the corresponding author upon reasonable request.

## Abbreviations

ELISA: enzyme-linked immunosorbent assay
N. caninum: Neospora caninum
OD: optical density
